# Autonomic cardiovascular control changes in recent heart transplant recipients lead to physiological limitations in response to orthostatic challenge and isometric exercise

**DOI:** 10.1007/s00421-019-04207-5

**Published:** 2019-08-12

**Authors:** Sissel Nygaard, Anders Haugom Christensen, Katrine Rolid, Kari Nytrøen, Lars Gullestad, Arnt Fiane, Erik Thaulow, Gaute Døhlen, Kristin Godang, J. Philip Saul, Vegard Bruun Bratholm Wyller

**Affiliations:** 1 Department of Pediatric Cardiology, Oslo University Hospital, Rikshospitalet, Oslo, Norway; 2 Faculty of Medicine, University of Oslo, Oslo, Norway; 3 Department of Cardiology, Oslo University Hospital, Rikshospitalet, Oslo, Norway; 4 KG Jebsen Center for Cardiac Research, University of Oslo, Oslo, Norway; 5 Center for Heart Failure Research, Oslo University Hospital, Oslo, Norway; 6 Department of Cardiothoracic Surgery, Oslo University Hospital, Rikshospitalet, Oslo, Norway; 7 Section of Specialized Endocrinology, Department of Endocrinology, Oslo University Hospital, Oslo, Norway; 8 Department of Pediatrics, West Virginia University, Morgantown, USA; 9 Department of Paediatrics, Akershus University Hospital, 1478 Lørenskog, Norway

**Keywords:** Heart transplantation, Autonomic cardiovascular control, Catecholamines, Denervation

## Abstract

**Purpose:**

Heart transplantation causes denervation of the donor heart, but the consequences for cardiovascular homeostasis remain to be fully understood. The present study investigated cardiovascular autonomic control at supine rest, during orthostatic challenge and during isometric exercise in heart transplant recipients (HTxR).

**Methods:**

A total of 50 HTxRs were investigated 7–12 weeks after transplant surgery and compared with 50 healthy control subjects. Continuous, noninvasive recordings of cardiovascular variables were carried out at supine rest, during 15 min of 60° head-up tilt and during 1 min of 30% of maximal voluntary handgrip. Plasma and urine catecholamines were assayed, and symptoms were charted.

**Results:**

At supine rest, heart rate, blood pressures and total peripheral resistance were higher, and stroke volume and end diastolic volume were lower in the HTxR group. During tilt, heart rate, blood pressures and total peripheral resistance increased less, and stroke volume and end diastolic volume decreased less. During handgrip, heart rate and cardiac output increased less, and stroke volume and end diastolic volume decreased less. Orthostatic symptoms were similar across the groups, but the HTxRs complained more of pale and cold hands.

**Conclusion:**

HTxRs are characterized by elevated blood pressures and total peripheral resistance at supine rest as well as attenuated blood pressures and total peripheral resistance responses during orthostatic challenge, possibly caused by low-pressure cardiopulmonary baroreceptor denervation. In addition, HTxRs show attenuated cardiac output response during isometric exercise due to efferent sympathetic denervation. These physiological limitations might have negative functional consequences.

## Introduction

Heart transplantation (HTx) remains the treatment of choice for end-stage heart failure, offering improved survival and quality of life for the recipients (Lund et al. 2017; Alraies and Eckman 2014). Normally, the heart as well as peripheral vessels are intimately controlled by the autonomic nervous system, ensuring immediate compensatory responses to all homeostatic aberration, which occurs—for instance—during orthostatic challenge and isometric exercise. However, cardiac transplantation results in surgical denervation of the donor heart, with complete loss of both efferent and afferent autonomic connections.

Denervation results in altered cardiovascular control and performance, including impaired cardiovascular reflex responses (Banner et al. 1990; Idema et al. 1994; Doering et al. 1996; Raczak et al. 1999; Awad et al. 2016). Although the functional consequences of these alterations have not been thoroughly investigated, denervation might contribute importantly to several important phenomena among HTx recipients (HTxRs). First, a tendency towards hypertension and peripheral vasoconstriction is well known among HTxRs (Idema et al. 1994; Bennett and Ventura 2017), but the underlying mechanisms remain to be fully understood, including whether there is a causal link between denervation and hypertension development. Second, being in an upright position might conceivably be difficult for HTxRs, as the baroreceptor reflex-mediated increase in heart rate during transition from supine to upright position is absent in a denervated heart. Still, symptoms of orthostatic intolerance do not appear to be more common among HTxRs than healthy controls; however, whether this is due to enhanced peripheral vessel responses has not been well addressed (Banner et al. 1990; Doering et al. 1991, 1996; Fitzpatrick et al. 1993). Third, HTxRs show reduced exercise capacity (Nytrøen and Gullestad 2013), but it is not known to what extent factors other than attenuated heart rate acceleration and myocardial contractility might contribute to this phenomenon; in particular, the potential role of altered peripheral vessel responses during exercise has not been focused on in previous reports.

Cardiovascular responses to orthostatic challenge have been extensively studied in healthy adults (Dambrink and Wieling 1987; Sprangers et al. 1991; Toska and Walloe 2002). The autonomic reflex adjustments during transition to upright position are characterized by altered afferent signaling from low-pressure cardiopulmonary baroreceptors as well as high-pressure arterial baroreceptors, normally resulting in increased heart rate due to enhanced sympathovagal balance to the heart as well as increased total peripheral resistance due to enhanced peripheral sympathetic activity. Cardiovascular responses to isometric exercise, on the other hand, are not dependent on afferent information from the heart, but are mainly due to afferent signaling from the working muscle combined with a “central command” in the brain (Kamiya et al. 2000). This results in a gradual increase in the set point of the barostat, normally enhancing sympathetic nervous activity to the heart as well as peripheral vasculature.

In a study of HTxRs, an experimental approach in which participants undergo both an orthostatic test (head-up tilt) and an isometric exercise test (handgrip) might be beneficial, as this combined setup might differentiate between effects of afferent and efferent cardiac denervation. Orthostatic responses are presumably affected by both afferent denervation of low-pressure cardiopulmonary baroreceptors and efferent denervation of the sinoatrial node and myocardium, whereas isometric exercise responses are affected by efferent denervation only. To the best of our knowledge, no previous study of HTxRs has applied a similar experimental approach. In addition, previous reports of orthostatic responses (Banner et al. 1990; Doering et al. 1991, 1996; Fitzpatrick et al. 1993) and isometric exercise responses (Roca et al. 1991; Brunner-La Rocca et al. 1998) in HTxRs are hampered by a small number of participants.

Thus, the aim of the present study was to investigate cardiovascular autonomic control at supine rest, during orthostatic challenge and during isometric exercise in a relatively large group of HTxRs and compare them to healthy control subjects. In particular, the study was designed to delineate the characteristics of peripheral vascular responses in HTxRs. We hypothesized that HTxRs would have increased total peripheral resistance during supine rest, enhanced total peripheral resistance responses to orthostatic challenge and unaltered responses to isometric exercise.

## Materials and methods

### Design

This study is part of the AccHEART project (Autonomic Cardiovascular Control after Heart Transplantation; ClinicalTrials ID: NCT01759966), which addresses autonomic denervation and reinnervation in a population-based prospective cohort of HTxRs. The present study reports results from the first encounter (baseline) only, compared with a group of healthy controls. AccHEART has been approved by the Norwegian National Committee for Ethics in Medical research. Participation was based upon informed consent.

### Participants

All patients at the Department of Cardiology, Oslo University Hospital receiving a heart transplant between January 2013 and December 2015 were screened for eligibility. To be included, we required the age of the recipient to be between 16 and 70 years and the transplant surgery to have been performed during the last 7–12 weeks (Table 1). This time window was chosen to ensure no interference from cardiovascular, neuroendocrine and inflammatory responses related to the surgical procedure per se, and at the same time avoid any reinnervation processes to have taken place. Exclusion criteria included dysfunction of the allograft, other chronic medical conditions, ECG abnormalities, other acute medical complications, and non-compliance. Drug usage was not considered an exclusion criterion; all eligible patients received immunosuppressive therapy (cyclosporine A/tacrolimus/everolimus, mycophenolate mofetil, corticosteroids) and statins, and the majority also received cardiovascular pharmaceuticals.

**Table 1:** Criteria for inclusion and exclusion in AccHeart

	Inclusion criteria	Exclusion criteria
Heart transplant recipients (HTxR)	Completed heart transplantation during the last 7–12 weeks Age > 16 years and < 70 years	Peri- or postoperative complications causing permanent dysfunction of the allograft (such as hyperacute rejection episodes, severe myocardial ischemia, etc.) Other chronic medical conditions, such as: Diabetes with HbA1C > 7.5% (mean value during 6 months prior to HTx) and/or manifest diabetic complications Renal failure with plasma creatinine > 200 µmol/l ECG abnormalities (scattered ectopic beats and minor conduction problems are allowed) Medical instability, non-compliance, multiorgan recipients, active infection or permanently bed-ridden patients
Healthy controls	Age and sex matching the patients	Other chronic diseases (such as diabetes mellitus) Permanent use of pharmaceuticals (including hormone drugs) Pregnancy

In addition, a volunteer group of healthy controls having the same distribution of sex and age as the HTxR group was recruited from the hospital staff and the general community population.

### Investigational program

All participants attended a 3-day investigational program at the Oslo University Hospital, Norway. The program was executed between 7.30 and 11.00 a.m. and was carried out in a fixed sequence. It included a clinical examination, autonomic cardiovascular control assessment, blood, and urine sampling, and questionnaire charting. All participants were instructed to abstain from tobacco products and caffeine 48 h prior to attendance, and to fast overnight. They were maintained on immunosuppressive medications, while all other drugs were paused on the morning of testing. They brought morning spot urine in a sterile container, and were instructed to apply a local anesthetic ointment (EMLA^®^, AstraZeneca) on both antecubital areas 1 h before arriving.

### Autonomic cardiovascular assessment

The Task Force Monitor^®^ (Model 3040i, CNSystems Medizintechnic, Graz, Austria) is a combined hardware and software device for noninvasive continuous recording of cardiovascular variables (Fortin et al. 2006). In the present study, recordings were performed (1) during 5-min supine rest; (2) during 15 min of 60° head-up tilt; (3) during 60 s of 30% of maximal voluntary handgrip. The handgrip procedure was repeated twice.

The duration of orthostatic challenge in the tilt procedure was shorter than what is usual in clinical settings (15 vs 30 min), to avoid syncope and other manifestations of failing cardiovascular homeostasis. Prior to initiation of the autonomic tests, participants were asked to perform maximum left-sided handgrip for 10 s, using an electronic device that provides a continuous display of the force (GRIP-IT s/n: 120521, Load Indicator System AB, Askim, Sweden). Based on the maximum value, the 30% level was calculated, and the participants used a couple of minutes to familiarize with this force.

Instantaneous heart rate (HR) was obtained from the electrocardiogram. Continuous arterial blood pressure was measured noninvasively beat-to-beat by finger plethysmography (Parati et al. 1989). The finger blood pressure values were automatically calibrated every fifth minute against conventional oscillometric upper arm measurements of arterial blood pressure. Impedance cardiography with electrodes placed on the neck and lower thorax was used to obtain a continuous recording of the temporal derivate of the transthoracic impedance (d*Z*/d*t*) (Denniston et al. 1976).

All primary cardiovascular variables (beat-to-beat recordings) were manually inspected and artifacts (such as non-sine node beats) were removed. Thereafter, beat-to-beat stroke volume (SV) was calculated from the impedance signal. Cardiac output (CO) was calculated as SV times HR, and total peripheral resistance (TPR) was calculated as mean blood pressure divided by CO. Flow-dependent variables were indexed according to body surface area (BSA), estimated from the formula BSA = 0.0235 × height (cm)^0.42246^ × weight (kg)^0.51456^.

For each individual, the median values of all cardiovascular variables were computed in the following epochs: (1) from 30 to 270 s. after start of supine rest; (2) from 30 to 270 s. after 60° head-up tilt; and (3) from 57 to 60 s. After initiation of each handgrip procedure, the mean value across the two most representative series of cardiovascular recordings (based on manual inspection) were used in subsequent analyses.

The epochs were defined in accordance with previously established routines at our laboratory (Wyller et al. 2007, 2008). For supine rest and tilt, the epochs correspond to the approximate steady-state situations before and after the application of orthostatic challenge, avoiding periods of mentally evoked autonomic activity and reflexive autonomic adjustments immediately before and after infliction of orthostatic challenge. For handgrip, the short (3 s) epoch was chosen to obtain the most extreme (maximal) value in each individual.

The cardiovascular response to tilt and handgrip was defined as the delta values (i.e., tilt values—supine rest values and handgrip values—supine rest values, respectively). Finally, mean values across all individuals in each group (HTxRs and healthy controls) were computed.

### Laboratory assays

Blood samples for catecholamine analysis were obtained from venous puncture in vacutainer tubes treated with ethylene glycol tetra acetic acid and glutathione from Sigma-Aldrich (St. Louis, USA). They were immediately put on ice and centrifuged (2500 rpm, 10 min, 4 °C) within 15 min. Both plasma epinephrine and norepinephrine were analyzed by high-performance liquid chromography (HPLC) (Agilent Technologies, Santa Clara, CA, USA) with a reversed-phase C-18 column (Chromsystem, München, Germany) and electrochemical detector (Antec, Leyden Decade II SCC, Zoeterwoude, The Netherlands) using a commercial kit from Chromsystem. The intra- and inter-assay coefficients of variation (CV) were 3.9% and 10.8%, respectively. Urine samples for catecholamine analysis were acidified to pH ~ 2.5 after collections, and thereafter analyzed by the same HPLC system as for plasma catecholamines. The intra- and inter-assay CVs were 3.9 and 5.2%, respectively. N-terminal pro-brain natriuretic peptide (NT-pBNP) in plasma was assayed at the accredited laboratory at Oslo University Hospital, Norway using routine procedures.

### Questionnaire

The autonomic symptom profile (ASP) is a validated inventory for assessing autonomic symptoms (Suarez et al. 1999). A composite score reflecting orthostatic symptoms was constructed from 8 single items from the ASP, addressing experiences of dizziness in specific situations (such as rising suddenly from supine position, taking a shower, etc.). The total sum score is from 0 to 8; higher values reflect more pronounced orthostatic problems. In addition, complaints of pale and cold hands were charted on a 1–5 Likert scale. A few complementary questions addressing personal symptoms and demographic data were added.

### Other variables

Background clinical information regarding the HTxR group was obtained from patients’ medical records. Activity level (steps/day) was charted using the activPAL accelerometer device (PAL Technologies, Glasgow, Scotland) for 7 consecutive days (thus including weekend days in all individuals) (Grant et al. 2006). Supine blood pressures were measured by a standard oscillometric device. Cardiac ejection fraction was obtained from transthoracic echocardiography, using the Simpson biplane method.

### Statistics

Statistical analyses were carried out using SPSS statistical software (SPSS Inc., Chicago, IL, USA). Previously, studies with identical experimental setup have been conducted at our institution; in the present study, power estimation suggested that a total of 50 participants in each group would support a power of about 80% to detect a medium effect size (Cohen’s *d* ≈ 0.5). Results are presented with mean (standard deviation) or median (interquartile range) for continuous data, depending on distribution. Categorical data are reported with frequencies. Statistical tests of differences between HTxRs and healthy controls were performed applying Student *t* test, Mann–Whitney *U* test, Chi square test or Fisher’s exact test as appropriate. Relationships between selected variables within the HTxR group were explored by Pearson correlation analyses. A *p* value ≤ 0.05 was considered statistical significant, and all tests were carried out two-sided. As several variables are strongly intercorrelated, *p* values were not adjusted for test multiplicity.

## Results

**Fig. 1:**
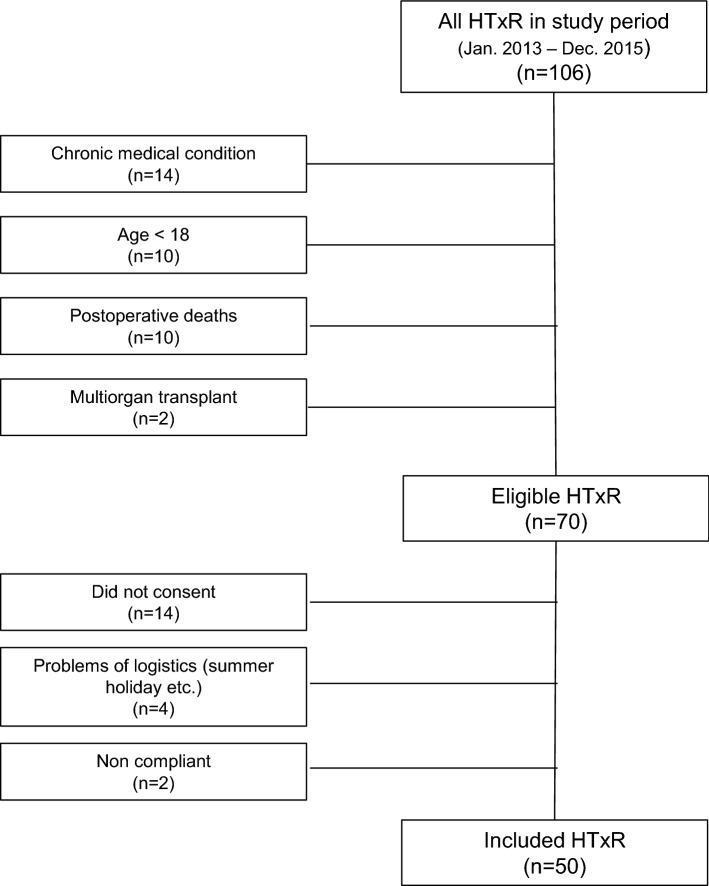
Flowchart of HTxR inclusion in AccHEART

A total of 106 patients received a heart transplant during the study period and was screened for eligibility in AccHEART. A total of 70 HTxRs were found to be eligible, of which 50 were finally enrolled after a mean (standard deviation) time of 2.5 (0.4) months after HTx surgery (Fig. 1). The remaining non-attending transplant recipients (*n* = 20) were comparable to the study participants regarding demographics and pre- and postoperative characteristics (Table 2).

**Table 2:** Characteristics of eligible heart transplant recipients during the study period^a^, included vs. not included

	Included in AccHEART	Not included in AccHEART	*p* value^b^
Number	50	20	n.a.
Sex—no. (%)			
Male	35 (70)	13 (65)	0.684
Female	15 (30)	7 (35)	
Ethnicity—no. (%)			
Norwegian	46 (92)	17 (85)	0.378
Not Norwegian	4 (8)	3 (15)	
Age—years, mean (SD)	48.2 (13.0)	47.9 (13.4)	0.924
Heart failure prior to HTx—years, mean (SD)	5.8 (5.5)	7.1 (4.5)	0.364
Waiting list for HTx—months, mean (SD)	3.1 (3.4)	4.1 (3.9)	0.305
Causes of heart failure—no. (%)			
Ischemic heart disease	13 (26)	7 (35)	0.495
Dilated cardiomyopathy	23 (46)	9 (45)	
Other	14 (28)	4 (20)	
Donor age—years, mean (SD)	36.4 (13.4)	42.4 (16.6)	0.120
Graft ischemic time—min, mean (SD)	172 (80)	145 (85)	0.229

*SD *standard deviation, *HTx* heart transplant

^a^From January 2013 until December 2015. All HTx recipients at the Oslo University Hospital (the only transplant center in Norway) were screened for eligibility in AccHEART

^b^Based upon Chi square test, Fisher’s exact test or Student *t* test, as appropriate

The included HTxRs underwent either primary single-organ orthotopic HT (*n* = 46) or re-transplantation (*n* = 4). The bicaval surgical technique with a small left atrial cuff and cavoatrial anastomoses was applied to all cases. They were maintained on immunosuppressive therapy, consisting of cyclosporine A (CsA) and mycophenolate mofetil in the majority of male recipients and tacrolimus and mycophenolate mofetil in the majority of female recipients. In patients with renal dysfunction (*n* = 13), a combination of low-dose CsA and the mTOR inhibitor everolimus was initiated. In addition, corticosteroids and statins were given to all HTxRs, and a total of 15 patients were on beta-blocking agents. Routine surveillance myocardial biopsies (each week during the first 8 weeks and then after 10 and 12 weeks) revealed an episode of acute rejection in a total of 14 HTxRs prior to inclusion in the AccHEART study. At the time of inclusion, acute rejection was revealed in five of the recipients (2R in 3 patients and 1R in 2 patients); all without symptoms and with normal heart function.

The HTxRs did not differ significantly from the healthy controls regarding sex, age, ethnicity and body mass index (Table 3). However, glomerular filtration rate and steps per day were lower, and HbA1c was higher in the HTxR group.

**Table 3:** Background characteristics of study participants

	HTx recipients	Healthy controls	*p* value^a^
Number	50	50	n.a.
Sex—no. (%)			
Male	35 (70)	35 (70)	1.000
Female	15 (30)	15 (30)	
Ethnicity—no. (%)			
Norwegian	46 (92)	48 (96)	0.678
Not Norwegian	4 (8)	2 (4)	
Age—years, mean (SD)	48.2 (13.0)	47.8 (12.4)	0.861
Body mass index—kg/m^2^, mean (SD)	24.8 (3.8)	25.2 (3.0)	0.586
Glomerular filtration rate, estimated—ml/min/1.73 m^2^, mean (SD)	55.8 (16.6)	92.2 (19.0)	**< 0.001**
HbA1c—%, mean (SD)	5.7 (0.7)	5.2 (0.3)	**< 0.001**
Steps per day—number, mean (SD)	5409 (2345)	8577 (3158)	**< 0.001**
Rejection episodes—no. (%)		n.a.	n.a.
No rejection	31.00 (62)		
Grade 1	11.00 (22)		
Grade 2 or more	8.00 (16)		

Numbers in bold indicate a *p* value < 0.05

*SD *standard deviation, *HTx *heart transplant

^a^Based upon Chi square test, Fisher’s exact test, or Student *t* test, as appropriate

Systolic blood pressure (measured supine using standard oscillometric technique) was slightly, but significantly increased in the HTxR group, whereas diastolic blood pressure was identical, as were left ventricular ejection fraction (Table 4). Levels of catecholamines were similar across groups, except for a slightly lower urine norepinephrine:creatinine ratio among HTxRs. Plasma NT-pBNP was significantly higher in the HTxR group, and showed a negative correlation with estimated glomerular filtration rate (Pearson’s *r* = − 0.34, *p* = 0.017). Orthostatic symptom score was identical across groups, whereas HTxRs complained more of pale and cold hands.

**Table 4:** Cardiovascular markers, neuroendocrine markers and symptoms in HTx recipients and healthy controls

	HTx recipients	Healthy controls	*p* value^a^	Adj. *p *value^b^
Cardiovascular markers				
Systolic blood pressure, supine—mmHg, mean (SD)	131.5 (14.9)	125.4 (14.6)	**0.040**	0.082
Diastolic blood pressure, supine—mmHg, mean (SD)	78.9 (8.4)	78.1 (9.1)	0.638	0.960
Left ventricular ejection fraction—%, mean (SD)	58.5 (5.4)	58.2 (4.6)	0.799	0.164
Neuroendocrine markers				
Plasma NT-pBNP—ng/l, median (IQR)	901 (926)	51 (66)	**< 0.001**	**< 0.001**
Plasma norepinephrine—pmol/l, median (IQR)	2072 (1844)	2041 (1139)	0.697	0.375
Urine norepinephrine:creatinine ratio—nmol/mmol, median (IQR)	9.0 (7.2)	12.2 (8.3)	**0.046**	0.178
Plasma epinephrine—pmol/l, median (IQR)	577 (405)	563 (373)	0.563	0.577
Urine epinephrine:creatinine ratio—nmol/mmol, median (IQR)	1.5 (1.2)	1.7 (1.2)	0.175	0.665
Symptoms				
Orthostatic symptoms—total score, mean (SD)	0.7 (0.8)	0.5 (0.8)	0.206	n.a.^c^
Pale and cold hands—score, mean (SD)	1.6 (1.2)	1.3 (0.9)	**0.038**	n.a.^c^

Numbers in bold indicate a *p* value < 0.05

*NT-pBNP *N-terminal pro-brain natriuretic peptide, *SD *standard deviation, *IQR *interquartile range, *HTx *heart transplant

^a^Based upon Chi square test, Fisher’s exact test, Student *t* test, or Mann–Whitney *U* test, as appropriate

^b^Adjusted according to group differences in activity levels (steps/day)

^c^Not applicable, as the statistical prerequisites for linear regression-based adjustment were not met

At supine rest during the autonomic experiments, heart rate, blood pressures and total peripheral resistance index were significantly higher, and stroke index and end diastolic volume index were significantly lower in the HTxR group compared with healthy controls (Table 5). Cardiac index was identical across the two groups. During tilt, heart rate, blood pressures and total peripheral resistance index increased less, and stroke index and end diastolic volume index decreased less in the HTxR group, whereas the cardiac index response did not differ. During handgrip, heart rate and cardiac index increased less, and stroke index and end diastolic volume index decreased less in the HTxR group, whereas the total peripheral resistance index response did not differ.

**Table 5:** Cardiovascular variables at supine rest, and responses to head-up tilt and handgrip in HTx recipients and healthy controls. Mean (95% confidence interval)

	Supine rest				Response to tilt, 60° head up				Response to handgrip, 30% max force			
	HTx recipients	Healthy controls	*p* value^a^	Adj. *p* value^b^	HTx recipients	Healthy controls	*p* value^a^	Adj. *p* value^b^	HTx recipients	Healthy controls	*p* value^a^	Adj. *p* value^b^
Heart rate—beats/min	81.6 (78.7 to 84.6)	56.0 (53.7 to 58.3)	**< 0.001**	**< 0.001**	1.9 (0.7 to 3.1)	14.6 (12.2 to 16.9)	**< 0.001**	**< 0.001**	− 0.3 (− 0.5 to − 0.1)	5.4 (3.9 to 7.0)	**< 0.001**	**< 0.001**
Systolic blood pressure—mmHg	117.9 (113.9 to 121.9)	109.0 (105.1 to 112.9)	**0.002**	**0.019**	5.8 (1.3 to 10.3)	11.2 (8.1 to 14.3)	**0.050**	0.117	15.1 (12.3 to 18.0)	17.0 (14.8 to 19.2)	0.307	0.144
Mean arterial blood pressure—mmHg	90.2 (87.2 to 93.2)	83.2 (80.1 to 86.3)	**0.002**	**0.020**	7.6 (3.7 to 11.4)	14.6 (11.9 to 17.3)	**0.003**	**0.018**	13.5 (10.9 to 16.2)	16.1 (14.0 to 18.2)	0.133	0.063
Diastolic blood pressure—mmHg	78.9 (76.0 to 81.7)	72.4 (69.4 to 75.3)	**0.002**	**0.024**	8.4 (5.0 to 11.9)	15.5 (12.7 to 18.2)	**0.002**	**0.013**	11.8 (9.1 to 14.5)	14.8 (12.6 to 17.0)	0.085	**0.046**
Stroke index—ml/m^2^	29.5 (27.8 to 31.1)	42.4 (40.0 to 44.7)	**< 0.001**	**< 0.001**	− 0.8 (− 1.9 to 0.4)	− 10.2 (− 12.3 to − 8.0)	**< 0.001**	**< 0.001**	0.3 (− 0.1 to 0.8)	− 1.7 (− 2.7 to − 0.8)	**< 0.001**	**0.018**
Cardiac index—l/min/m^2^	2.4 (2.3 to 2.5)	2.4 (2.2 to 2.5)	0.906	0.314	0.01 (− 0.09 to 0.10)	− 0.13 (− 0.28 to 0.02)	0.126	0.363	0.01 (− 0.02 to 0.05)	0.10 (0.03 to 0.17)	**0.025**	0.119
Total peripheral resistance index—mmHg/l/min/m^2^	10.8 (9.9 to 11.6)	9.2 (8.5 to 9.9)	**0.006**	**0.009**	0.9 (0.3 to 1.5)	1.8 (1.2 to 2.5)	**0.044**	0.199	1.5 (1.2 to 1.8)	1.3 (1.0 to 1.7)	0.536	0.748
End diastolic volume index—ml/m^2^	51.5 (49.0 to 54.0)	68.6 (65.0 to 72.1)	**< 0.001**	**< 0.001**	1.0 (− 1.2 to 3.1)	− 11.2 (− 14.6 to − 7.9)	**< 0.001**	**< 0.001**	0.1 (− 0.5 to 0.7)	− 2.5 (− 3.9 to − 1.1)	**0.001**	0.079

Numbers in bold indicate a *p* value < 0.05

*HTx *heart transplant

^a^Based upon Student *t* test. A Bonferroni-correction due to multiple statistical tests suggests a level of significance of 0.05/26 = 0.002

^b^Adjusted according to group differences in activity levels (steps/day)

Within the HTxR group, total peripheral resistance index during supine rest showed a strong negative correlation to body weight and to dosage of prednisolone; in partial correlation analyses controlling for body weight, the associations between prednisolone and TPRI became negligible and non-significant (Table 6). In addition, urine norepinephrine:creatinine ratio was positively associated with mean arterial blood pressure response during tilt (Table 6). HTxRs receiving diuretics tended to have lower supine mean arterial blood pressure, and an attenuated response of mean arterial blood pressure and total peripheral resistance index during tilt (Table 7).

**Table 6:** Correlations (Pearson’s *r*) between cardiovascular variables of interest and possible confounding factors within the HTx recipient group

	Supine: mean arterial blood pressure, mmHg		Supine: total peripheral resistance index, mmHg/l/min/m^2^		Tilt response: mean arterial blood pressure, mmHg		Tilt response: total peripheral resistance index, mmHg/l/min/m^2^		Handgrip response: cardiac index, l/min/m^2^	
	*r*	*p* value	*r*	*p* value	*r*	*p* value	*r*	*p* value	*r*	*p* value
Glomerular filtration rate, estimated, ml/min/1.73 m^2^	0.19	0.185	0.06	0.710	0.10	0.512	0.11	0.483	− 0.16	0.303
HbA1c, %	0.00	0.991	− 0.25	0.081	0.05	0.740	0.07	0.652	− 0.25	0.100
Steps per day, number	0.11	0.534	0.10	0.549	− 0.15	0.409	− 0.05	0.760	− 0.02	0.910
NT-pBNP, ng/l	0.04	0.793	− 0.02	0.877	− 0.22	0.147	− 0.12	0.428	0.07	0.644
Urine Norepinephrine:creatinine ratio, nmol/mmol	− 0.26	0.080	0.08	0.596	**0.33**	**0.028**	0.20	0.183	− 0.11	0.488
Cyclosporine dosage^a^, mg/day	0.20	0.255	− 0.05	0.787	0.05	0.767	0.10	0.557	0.10	0.604
Tacrolimus dosage^b^, mg/day	− 0.09	0.777	− 0.18	0.573	0.47	0.145	0.55	0.081	0.14	0.659
Prednisolone dosage^c^, mg/day	0.17	0.243	− **0.52**	**< 0.001**	− 0.20	0.193	− 0.12	0.443	− 0.07	0.652
Controlled for weight (partial corr.)			− 0.03	0.842						
Metoprolol dosage^d^, mg/day	0.05	0.870	0.38	0.196	− 0.24	0.457	− 0.28	0.382	− 0.30	0.343
Valganciclovir dosage^e^, mg/day	0.13	0.558	0.01	0.955	− 0.12	0.602	− 0.15	0.508	0.12	0.613

Numbers in bold indicate a *p* value < 0.05

*NT-pBNP *N-terminal pro-brain natriuretic peptide, *HTx *heart transplant

^a^Used by a total of 36 HTx recipients

^b^Used by a total of 13 HTx recipients

^c^Used by a total of 50 HTx recipients

^d^Used by a total of 14 HTx recipients

^e^Used by a total of 22 HTx recipients

**Table 7:** Cardiovascular variables of interest in subgroups of HTx recipients according to medication usage. Mean (95% confidence intervals)

	Diuretics^a^		Calcium blocker^b^		Betablocker^c^		All HTx recipients^d^	All healthy controls^d^
	Yes (*n* = 40)	No (*n* = 10)	Yes (*n* = 13)	No (*n* = 37)	Yes (*n* = 15)	No (*n* = 25)		
Supine: mean arterial blood pressure, mmHg	88.9 (85.6 to 92.3)	95.7 (89.3 to 102.1)	94.1 (89.5 to 98.6)	88.9 (85.2 to 92.6)	92.1 (86.6 to 97.6)	89.4 (85.7 to 93.1)	90.2 (87.2 to 93.2)	83.2 (80.1 to 86.3)
Supine: total peripheral resistance index, mmHg/l/min/m^2^	10.7 (9.7 to 11.7)	11.1 (10.0 to 12.3)	10.3 (8.9 to 11.7)	10.9 (9.9 to 12.0)	11.1 (9.0 to 13.3)	10.6 (9.7 to 11.5)	10.8 (9.9 to 11.6)	9.2 (8.5 to 9.9)
Tilt response: mean arterial blood pressure, mmHg	6.6 (2.6 to 10.6)	12.1 (− 1.3 to 25.4)	3.1 (− 3.0 to 9.1)	8.8 (4.2 to 13.5)	7.3 (0.5 to 14.1)	7.7 (2.8 to 12.5)	7.6 (3.7 to 11.4)	14.6 (11.9 to 17.3)
Tilt response: total peripheral resistance index, mmHg/l/min/m^2^	0.7 (0.02 to 1.3)	1.9 (− 0.01 to 3.9)	1.2 (− 0.3 to 2.6)	0.8 (0.1 to 1.5)	1.0 (− 0.1 to 2.0)	0.9 (0.1 to 1.7)	0.9 (0.3 to 1.5)	1.8 (1.2 to 2.5)
Handgrip response: cardiac index, l/min/m^2^	0.01 (− 0.03 to 0.05)	0.04 (− 0.08 to 0.17)	0.06 (− 0.03 to 0.16)	− 0.003 (− 0.04 to 0.4)	− 0.01 (− 0.08 to 0.06)	0.02 (− 0.02 to 0.07)	0.01 (− 0.02 to 0.05)	0.10 (0.03 to 0.17)

Numbers in bold indicate a *p* value < 0.05

*HTx* heart transplant

^a^Includes bumetanide (*n* = 37), furosemide (*n* = 2) and thiazide (*n* = 5)

^b^Includes nifedipine (*n* = 10) and amlodipine (*n* = 3)

^c^Includes metoprolol (*n* = 14) and carvedilol (*n* = 1)

^d^Shown for clarity

## Discussion

The most important results of this study are: (1) at supine rest, HTxRs have higher blood pressures and total peripheral resistance than healthy controls. (2) During orthostatic challenge, HTxRs have attenuated blood pressure and total peripheral resistance responses. (3) During isometric exercise, HTxRs have preserved blood pressure and total peripheral resistance responses, but an attenuated cardiac output response.

The tendencies towards hypertension and peripheral vasoconstriction in HTxRs are well known (Idema et al. 1994; Bennett and Ventura 2017). In the present study we also observed more frequent complaints of pale and cold hands in the HTxR group, suggesting a direct link between altered peripheral vasoconstriction and patient well-being. While the underlying mechanisms remain to be fully understood, a main causal factor might be the usage of immunosuppressive medication, in particular calcineurin inhibitors and corticosteroids (Idema et al. 1994; Bennett and Ventura 2017). For instance, cyclosporine (CyA) has been reported to increase circadian sympathetic activity (Scherrer et al. 1990), to interfere with pressor responses of vasoconstrictor hormones (Lustig et al. 1987), to alter the prostacyclin–thromboxane A2 balance in favor of vasoconstriction (Kahan 1989) and to induce vessel wall inflammation (Reeves et al. 1986). Interestingly, in the present study, there were no significant correlations between calcineurin inhibitors dosage and cardiovascular variables, and the association between glucocorticoid dosage and resting vasoconstriction disappeared when controlling for body weight. Thus, the tendencies towards hypertension and vasoconstriction in HTxRs might have other explanations. We speculate that the permanent denervation of low-pressure cardiopulmonary baroreceptors in the heart might be a contributing factor. Tonic stimulation of these receptors in the normal heart reflexively reduces sympathetic outflow to the peripheral vasculature (Triedman et al. 1993; Goldstein 2001); thus, a lack of afferent impulses would presumably cause a permanent enhancement of sympathetic vasomotion. This hypothesis should be scrutinized in further research.

The attenuated response of blood pressure and peripheral vasoconstriction to orthostatic challenge might at first seem surprising. Since arterial baroreceptors are preserved in HTxRs, we hypothesized that the inability to increase heart rate and cardiac output during orthostasis due to efferent cardiac denervation would lead to a compensatory response of total peripheral resistance, which would be larger than in healthy controls. The preserved vasoconstrictive response we found during handgrip suggests the efferent sympathetic pathways to peripheral vessels are intact. However, it is clear in the literature that low-pressure cardiopulmonary baroreceptors in the heart play an important role in modulating the peripheral sympathetic response to orthostasis, so the lack of afferent signals from these receptors during volume depletion might attenuate the response of efferent sympathetic outflow (Triedman et al. 1993; Goldstein 2001). The usage of diuretics among some HTxRs might add to this effect. Surprisingly, the substantial alterations of orthostatic homeostasis after HTx did not increase orthostatic symptoms. This is in line with previous findings and suggests that the subjective experiences of orthostatic intolerance might be more specifically related to certain phenomena, such as reflex-mediated (“vasovagal”) hypotension which presupposes normal cardiac innervation and, therefore, does not exist early after HTx.

The attenuated cardiac output response to handgrip in the HTxR group might be directly explained from cardiac sympathetic denervation, which inhibits the normal increase in heart rate as well as myocardial contractility during isometric exercise (Kamiya et al. 2000). In addition, in the present study, cardiac output was associated with end diastolic volume index (Pearson’s *r* = 0.78, *p* < 0.001), suggesting that usage of diuretics and thereby reduced diastolic filling might contribute to lower cardiac output response. The functional impact of this particular homeostatic aberration remains to be fully characterized, but reduced exercise capacity is a logical assumption. This hypothesis deserves further investigations; interestingly, handgrip strength is a marker of frailty in heart failure patients (Chung et al. 2014).

The elevated heart rate at supine rest as well as the lack of heart rate response under both experimental conditions in the HTxR group confirms complete efferent cardiac denervation, as assumed early after HTx (Robson et al. 1989; Doering et al. 1991; Awad et al. 2016). Cardiac sympathetic reinnervation over time has been demonstrated in previous reports, but the speed of reinnervation process, as well as the eventual presence of parasympathetic and afferent reinnervation, remain to be fully investigated and should be a focus of future research. The group differences in stroke volume and end diastolic volume are likely explained by the differences in heart rate and thereby diastolic filling time (Elstad et al. 2001); accordingly, heart rate, stroke index and end diastolic volume index were strongly intercorrelated in the present study.

Taken together, the findings reported in the present study are likely responsible in part for reduced exercise capacity in heart transplant recipients (Nytrøen and Gullestad 2013). Specifically, in addition to the well-recognized effects of the limited response of heart rate to buffering the effects of posture and increasing cardiac output during exercise in transplant patients, increased resting peripheral resistance and the subsequent inability to modulate resistance normally is also likely to affect both isometric and isotonic exercise responses. That said, level of activity has a well-known effect on autonomic cardiovascular control (O’Sullivan and Bell 2000). Thus, the lower number of steps per day in the HTxR group than the control group might be a cause for as well as an effect of autonomic alterations. Of note, group differences regarding total peripheral resistance response to orthostatic challenge and cardiac output response to isometric exercise became non-significant when *p* values were adjusted for differences in steps per day.

### Strengths and limitations

Strengths of the present study include the relatively large number of HTxRs as compared to other studies in the field, and the population-based nature of patient recruitment from a nationwide transplant center. We cannot totally exclude selection bias, but the similarity of baseline characteristics between the included and non-included group of eligible patients suggests wide generalizability of the results. The time window for investigations of the HTxRs (7–12 weeks after transplant surgery) was chosen to avoid interference from compensatory responses related to the surgical procedure per se, and at the same time avoid any reinnervation processes to have taken place. While the data suggest that the latter presumption holds, we cannot completely rule out that long-lasting effects from the surgical procedure and subsequent intensive care might have influenced the results. Cardiac transplantation is an effective treatment against chronic heart failure; as the patient group was investigated 7–12 weeks after transplant surgery, we find it likely that previous autonomic abnormalities related to a failing heart would have normalized prior to inclusion. However, it is possible that “hangover” effects from the patients’ previous health condition or another underlying disease process might have biased the results.

Missing data were not imputed; however, the numbers of missing data points were few and considered negligible. *p* values were not adjusted for test multiplicity; however, the computed confidence intervals support the conclusions drawn from the statistical tests (i.e., the central estimate of one group is not included in the confidence interval for the central estimate of the other group when a statistically significant difference is suggested from the *p* value).

## Conclusion

HTxRs are characterized by elevated blood pressures and total peripheral resistance at supine rest, as well as attenuated blood pressure and total peripheral resistance responses during orthostatic challenge; both these effects might be caused by low-pressure cardiopulmonary baroreceptor denervation. In addition, HTxRs show attenuated cardiac output response during isometric exercise due to efferent sympathetic denervation. These physiological limitations might have negative functional consequences in HTxRs.

## Data Availability

The datasets generated during and/or analyzed during the current study are available from the corresponding author on reasonable request.

## Abbreviations

AccHEART: Autonomic cardiovascular control after heart transplantation
ANS: Autonomic nervous system
ASP: Autonomic symptom profile
CO: Cardiac output
CsA: Cyclosporine A
CV: Coefficient of variation
HPLC: High-performance liquid chromography
HR: Heart rate
HTxR: Heart transplant recipients
NT-pBNP: N-terminal pro-brain natriuretic peptide
SV: Stroke volume
TPR: Total peripheral resistance
